# Outcomes comparison between fibula and DCIA free flaps in head and neck reconstructive surgery: Implications for donor site selection

**DOI:** 10.4317/medoral.28000

**Published:** 2026-01-24

**Authors:** Mario Guerrero-Martín, Fernando Almeida-Parra, Patricia Guerrero-Martín, Víctor Vega-Barreto, Julio Acero-Sanz

**Affiliations:** 1 Oral and Maxillofacial Surgery Department, Ramon y Cajal and Puerta de Hierro University Hospitals, Madrid, Spain; 2 Ramon y Cajal Health Research Institute (IRYCIS), University of Alcala, Madrid, Spain; 3 IMF Smart Education, University of Avila, Avila, Spain; 4 Doctoral Programme in Health Sciences, Doctoral School, University of Alcala, Alcala de Henares, Madrid, Spain

## Abstract

**Background:**

Donor site selection for bone tissue reconstruction in head and neck surgery has remained a matter of debate since the advent of microsurgical techniques. Both the fibula and the deep circumflex iliac artery (DCIA) free flaps offer specific advantages but also present distinct morbidity profiles that must be considered to provide optimized and personalized reconstructive treatment for each patient.

**Material and Methods:**

A retrospective morbidity analysis was conducted on 66 osseous free flaps (fibula and DCIA) used for head and neck reconstructive surgery at Ramón y Cajal University Hospital over a 6-year period (2018-2024). Surgical variables, as well as local and systemic complications, were analyzed.

**Results:**

Reconstruction with the DCIA free flap was associated with a threefold higher risk of developing systemic infections not related to surgical site (47.37% vs. 21.28%; OR=3.33; 95% CI=1.07-10.41) compared with the fibula free flap. No statistically significant differences were observed between the two groups regarding free flap failure, surgical site infections, postoperative bleeding, microvascular complications, or donor site morbidity.

**Conclusions:**

the DCIA free flap may be associated with a higher risk of systemic infections. Therefore, for patients with predisposing factors for systemic infections, the fibula free flap might represent a safer donor site. These findings could help refine donor site selection and improve individualized planning in head and neck reconstructive surgery.

## Introduction

The primary goal of head and neck reconstructive surgery is to restore both function and aesthetics of the affected anatomical region. To achieve this objective, microvascular free flaps have become the current gold standard. A free flap is a composite tissue segment with an independent blood supply, harvested from a donor site and transferred to repair a defect. Microsurgical techniques are required to ensure tissue viability at the recipient site. These flaps can include different tissue types, with osseous flaps being of particular relevance for craniomaxillofacial and reconstructive surgeons (1 - 3).

Concerning osseous options, the fibula free flap (FFF) and the deep circumflex iliac artery (DCIA) free flap are the most commonly used in head and neck reconstruction. The fibula free flap was first introduced by Hidalgo in 1989 for mandibular reconstruction. The fibula provides a long segment of dense cortical bone (22-25 cm) while preserving 6-7 cm proximal and distal bone. Its vascular pedicle consists of the peroneal artery and its comitant veins. Owing to the generous amount of available bone, the fibula flap is considered the flap of choice for extensive defects, such as those resulting from total or subtotal mandibulectomy (2 , 4).

The DCIA free flap, described by Urken in 1989 for oromandibular reconstruction, is based on the deep circumflex iliac artery and its comitant veins, branches of the external iliac vessels. The iliac crest provides up to 12-14 cm of cortico-cancellous bone with abundant marrow between two thin cortical plates. Given the limited bone stock, the DCIA flap is particularly suitable for shorter defects, such as those involving the mandibular body or angle (2 , 4).

Both flaps can be harvested with associated soft tissue, including muscle and/or skin paddles, provided that perforator vessels are preserved to ensure viability (2).

While the choice of donor site primarily depends on the characteristics of the defect-amount of bone and soft tissue required-patient comorbidities must also be considered. The present study aimed to evaluate whether the choice of donor site-fibula or DCIA-could affect postoperative morbidity in head and neck reconstruction, with the goal of optimizing and individualizing flap selection for each patient.

## Material and Methods

This study is a retrospective observational analysis of 66 osseous free flaps performed in 64 patients who underwent head and neck reconstructive surgery at Ramón y Cajal University Hospital between 2018 and 2024.

Demographic variables (age and gender), comorbidities, cause of reconstruction, and postoperative complications (systemic, donor site, recipient site) were collected. The data were used to conduct a comparative morbidity analysis between the fibula and deep circumflex iliac artery free flaps groups.

Statistical analysis

Descriptive statistics were applied to characterize the sample in terms of gender, age, pre-existing comorbidities (prior radiotherapy and diabetes mellitus), etiology of reconstruction, and anatomical site of the defect.

Inferential analysis was performed to compare outcomes between groups. Odds ratios (ORs) with 95% confidence intervals (CIs) were calculated to determine whether the DCIA flaps represented a risk factor, compared with the fibula flap, for developing postoperative complications including flap failure, surgical site infection, systemic infection, postoperative bleeding and microvascular events. Statistical significance was assumed when the null value (OR=1) was not included within the 95% CI.

Welch's t-test was used to compare mean age between groups, and Fisher's exact test was applied to assess differences in the proportion of patients with prior radiotherapy. A p-value &lt;0.05 was considered statistically significant. All analyses were performed using Python statistical software.

## Results

Out of 66 osseous free flaps performed, 35 (53%) were performed in male patients and 31 (47%) in female patients. The FFF was the most commonly used donor site (47 cases), whereas the DCIA free flap was performed in 19 cases. The fibula flap was slightly more common in female patients (51.1%) than in males (48.9%), while the DCIA flap showed the opposite trend being more frequent in males (63.2%) than in females (36.8%).

The mean age of the entire cohort was 58.6±14.8 years. Patients reconstructed with the fibula flap were slightly older (mean=59.0±15.2 years) than those reconstructed with the DCIA flap (mean=56.5±14.2 years), although this difference was not statistically significant (p=0.51).

Regarding the reconstruction site, FFF was used for maxilla in 10 cases (21.3%) and for the mandible in 37 (78.7%). DCIA flaps were applied for the maxilla in 2 cases (10.5%) and for the mandible in 17 (89.5%).

Mandibular defects were classified according to HCL classification of Jewer and Boyd and maxillary defects were classified according to Brown's classification. In the FFF group, reconstructed mandibular defects were classified as: Type L in 13 cases, type LCL in 13 cases, type H in 5 cases, type LC in 2 cases, type C in 2 cases, type HC in 1 case and type HCL in 1 case. In the DCIA group, reconstructed mandibular defects were type L in 10 cases, type LCL in 3 cases, type C in 3 cases and type H in 1 case.

Regarding maxillary defects, FFF was used to repair 3 Brown type IIb defects, and one each of the following Brown types: Ib, IIa, IIc, IId, IIIb, IIId and IVa. The DCIA flap was used to repair 2 Brown type IIId defects.

In the fibula group, 35 defects (74.5% of FFF group) involved soft tissue, while in the DCIA group, 12 defects involved soft tissue (63.2% of DCIA group).

An osteomyocutaneous flap was harvested in 42 FFF cases (89.4% of FFF group), whereas none was harvested in the DCIA group. Conversely, an osteomuscular flap was harvested in 5 FFF cases (10.6% of FFF group) and in all DCIA cases.

The etiology of reconstruction was tumor resection in 55 cases (83.3%) and non-oncologic in 11 cases (16.7%). Among oncologic cases, squamous cell carcinoma represented the predominant diagnosis (83.6%). The distribution of oncologic indications was similar between groups: 39 FFFs (83%) and 16 DCIA flaps (84.2%). Oncologic reconstruction was immediate in 35 FFFs (89.7%) and in 13 DCIA flaps (81.3%), whereas it was delayed in 4 FFFs (10.3%) and in 3 DCIA flaps (18.7%).

Comorbidities included diabetes mellitus in 6 patients (9.1%), all of whom were reconstructed with fibula flaps, and prior head and neck radiotherapy in 18 patients (27.3%). Fifteen of these underwent FFF reconstruction (31.9%) and three had DCIA reconstruction (15.8%); this difference was not statistically significant (p=0.19) (Table 1).

Table 1

**Figure 1:**
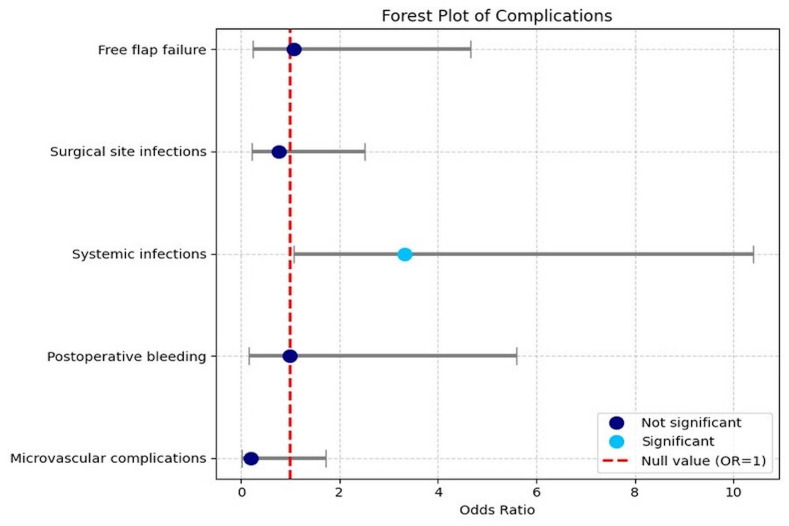
Forest plot: Postoperative complications.

Regarding the complications observed in the sample (Figure 1).

1

Flap failure: 10 out of 66 flaps (15.2%) failed, 7 fibula (14.9% of FFF) and 3 DCIA (15.8% of DCIA). No significant difference was found (OR=1.07; 95% CI 0.25-4.67).

Surgical site infection: This occurred in 20 patients (30.3%); 15 in the fibula group (31.9%; 13 in neck wound, 1 intraoral, 1 in donor site and neck wound) and 5 in the DCIA group (26.3%; 2 in the neck wound, 2 in the donor site, 1 in the tracheostomy wound). The difference was not statistically significant (OR=0.76, 95% CI 0.23-2.51).

Systemic infection: 19 patients (28.8%) developed systemic infections, 10 in the fibula group (21.3%) and 9 in the DCIA group (47.4%). The difference was statistically significant, indicating a threefold higher risk with the DCIA flap (OR=3.33, 95% CI 1.07-10.41). The most common type was respiratory infection (69.7%).

Postoperative bleeding: 7 patients (10.6%) experienced bleeding, 5 after fibula (10.6%, 4 in the neck and 1 intraoral) and 2 after DCIA (10.5%, both in the neck) reconstruction. No significant difference was observed (OR=0.99, 95% CI 0.17-5.60).

Microvascular complications: These were observed in 11 cases (16.7%); 10 after fibula (21.3%, 7 with venous thrombosis, 1 with venous dehiscence, 1 with arterial thrombosis and 1 with arterio-venous thrombosis) and 1 after DCIA (5.3%, 1 with venous thrombosis) reconstruction, without significant difference (OR=0.21, 95% CI 0.02-1.73). Salvage surgery was successful in 8 out of 11 cases (all in the fibula group).

## Discussion

Reconstruction of craniomaxillofacial defects requiring osseous tissue continues to be a complex and technically demanding endeavor, in which selecting the most appropriate donor site remains essential. The FFF is widely regarded as the workhorse for maxillomandibular reconstruction and is employed more frequently than the DCIA flap. This trend was also observed in our cohort, where FFF accounted for 71.2% of reconstructions (4 , 5).

Mashrah et al. conducted a network meta-analysis comparing the survival rates of vascularized osseous flaps used in mandibular reconstruction and found no significant difference between fibula and DCIA flaps. These results are consistent with our findings, which demonstrated survival rates of 85.1% for FFF and 84.2% for DCIA flaps. Therefore, both flaps represent reliable and valuable donor sites for head and neck reconstruction. The survival rate of FFF in our database is consistent with published literature reports, where rates vary from 45% to 100% across different series. To our knowledge, there is no literature comparing DCIA flap survival rate between several observational studies. Both flaps provide a long vascular pedicle, the option to include a skin paddle (however, the skin paddle in DCIA flaps are less useful for intraoral reconstruction due to greater bulkiness and reduced pliability compared with those of fibula flaps) and sufficient bone stock to restore maxillomandibular defects (the iliac crest bone being more similar in height and width to the native mandible than that of the fibula, but for extensive defects, such as total or subtotal mandibulectomy, fibula flaps remain the only osseous flaps to restore the bone defect) (4 , 6).

Regarding donor site-related drawbacks, FFF may be contraindicated in cases of atherosclerotic vessel disease (which must be excluded during preoperative assessment) and are associated with delayed ambulation, whereas the DCIA flaps are limited by smaller bone stock and the restricted mobility of the soft tissue component (4).

No significant differences were observed in surgical site infection rates (donor and recipient sites) between FFF and DCIA reconstructions (OR=0.76; 95% CI 0.23-2.51). The majority of infections occurred in the cervical wound, with only three donor-site infections recorded (1 in the fibula group and 2 in the DCIA group). These results are consistent with the previously published data indicating no difference in donor-site morbidity between the two techniques (8).

A key finding of our study was the substantially higher rate of systemic infections in patients reconstructed with DCIA flaps (OR=3.33; 95% CI 1.07-10.41). This association has not been previously reported in the literature, to the best of our knowledge. The majority of these infections were respiratory in origin (69.7%). We hypothesize that restricted trunk flexion and limited mobility following DCIA flap harvesting, attributable to postoperative pain, may predispose patients to bronchoaspiration of oropharyngeal flora and subsequent respiratory colonization. In terms of postoperative pain, restrictive mechanisms may also be involved. Furthermore, this risk could be compounded by dysfunction in swallowing and airway protection, which often accompany extensive maxillomandibular resections. A previous study conducted within the same healthcare system reported a higher systemic infection rate (50.8%), which was also predominantly respiratory in nature. However, that cohort included mainly soft-tissue free flaps, thereby limiting direct comparison (9).

Postoperative bleeding rates did not differ significantly between groups (OR=0.99; 95% CI 0.17-5.60) at the recipient site. None occurred at the donor site. Some authors have suggested that DCIA flap may carry an increased bleeding risk at the donor site due to the rich vascularization of the iliac crest, although our findings do not support this hypothesis (8 , 10).

Similarly, no significant differences were observed in microvascular complication rates between the two donor sites. Although FFF exhibited a higher numerical rate (21.3% vs. 5.3%), this difference was not statistically significant. Venous thrombosis was the most common event, consistent with prior literature. The slightly higher rate in the FFF group may reflect the greater prevalence of peripheral vascular disease in lower-limb vessels compared with pelvic circulation (4).

## Conclusions

In summary, the DCIA free flap was identified as a risk factor for systemic infection compared with the fibula flap in this cohort. Consequently, the fibula flap should be preferred in patients with an increased susceptibility to infection, such as elderly or immunocompromised individuals.

The main strengths of this study include that all procedures were performed by the same experienced surgical team at a tertiary referral center for craniomaxillofacial reconstruction, ensuring homogeneity in surgical technique and postoperative care. The principal limitations are its retrospective, single-center design and the relatively limited sample size.

Future investigations should aim to validate these findings through multicenter prospective studies and randomized clinical trials, which are required to establish robust, evidence-based recommendations for donor site selection in osseous free flap reconstruction.

## Figures and Tables

**Table 1:** Table Descriptive comparison of the two subgroups (FFF and DCIA free flap).

	FFF (n=47)	DCIA free flap (n=19)
Gender	Male 48.9%Female 51.1%	Male 63.2%Female 36.8%
Mean age (years)	58.96	56.53
Oncologic reconstruction	83%	84.2%
Reconstructed anatomical site	Maxilla 21.3%Mandible 78.7%	Maxilla 10.5%Mandible 89.5%
Diabetes mellitus	12.8%	0%
Prior radiotherapy	31.9%	15.8%

1

## Data Availability

The data supporting the findings of this study are available upon reasonable request.
